# Roles for Human Papillomavirus Type 16 L1 Cysteine Residues 161, 229, and 379 in Genome Encapsidation and Capsid Stability

**DOI:** 10.1371/journal.pone.0099488

**Published:** 2014-06-11

**Authors:** Eric J. Ryndock, Michael J. Conway, Samina Alam, Sana Gul, Sheeba Murad, Neil D. Christensen, Craig Meyers

**Affiliations:** 1 Department of Microbiology and Immunology, The Pennsylvania State University College of Medicine, Hershey, Pennsylvania, United States of America; 2 Department of Pathology, The Pennsylvania State University College of Medicine, Hershey, Pennsylvania, United States of America; 3 Health Care Biotechnology Department, Atta-ur-Rahman School of Applied Biosciences, National University of Science and Technology, Islamabad, Pakistan; International Centre for Genetic Engineering and Biotechnology, Italy

## Abstract

Human papillomavirus (HPV) capsids are formed through a network of inter- and intra-pentameric hydrophobic interactions and disulfide bonds. 72 pentamers of the major capsid protein, L1, and an unknown amount of the minor capsid protein, L2, form the structure of the capsid. There are 12 conserved L1 cysteine residues in HPV16. While C175, C185, and C428 have been implicated in the formation of a critical inter-pentameric disulfide bond, no structural or functional roles have been firmly attributed to any of the other conserved cysteine residues. Here, we show that substitution of cysteine residues C161, C229, and C379 for serine hinders the accumulation of endonuclease-resistant genomes as virions mature within stratifying and differentiating human epithelial tissue. C229S mutant virions form, but are non-infectious. These studies add detail to the differentiation-dependent assembly and maturation that occur during the HPV16 life cycle in human tissue.

## Introduction

HPV capsids are composed of 360 copies of the major capsid protein, L1, and an unknown amount of the minor capsid protein, L2 [1]. L1 proteins rapidly assemble into pentamers through hydrophobic interactions [2], [3]. The 72 pentamers that form the capsid are linked together through a complex network of hydrophobic interactions and disulfide bonds [4]–[13]. Capsids contain a single, circular, dsDNA genome of approximately 8 kb, which associates with histones to form a chromatin-like structure [7], [14]. The genome increases the stability of the capsid, increasing its resistance to environmental stresses such as proteolysis [14].

We showed previously that native HPV16 virions exploit a tissue-spanning redox gradient that facilitates assembly and maturation events in the context of the complete papillomavirus life cycle [15]–[17]. Further, substitution of cysteine residues C175, C185, and C428 for serine led to a late-stage defect in the accumulation of endonuclease-resistant genomes and made these mutant virions more fragile than wild-type virions. This evidence is similar to studies using virus-like particles (VLPs), recombinant HPV particles made in monolayer cell systems, that also show an importance for C175, C185, and C428 in capsid stability [18]. In this study, we determined if other conserved L1 cysteine residues were involved in the packaging of endonuclease-resistant genomes, and if they similarly affected the stability of HPV16 capsids.

It has been shown that mutation of C161, C229, and C379 in HPV16 VLPs causes a decreased resistance to proteolysis, highlighting an importance for these cysteines in capsid structure [18]. However, analysis of the crystal structure of a small, 12-pentamer L1-only HPV16 VLP and a high-resolution cryo-EM image of native BPV1 suggest that disulfide bonds between these residues do not exist [19], [20]. Additionally, their positions do not appear close enough to facilitate disulfide bonding without gross structural changes of the capsid. However, Simian virus 40 (SV40) has been shown to require cysteines that are not involved in inter-pentameric disulfide bonding in the final structure of stable and infectious virions, specifically, they are important during early steps of VP1 folding and oligomerization and virion assembly by forming transient disulfide bonds [21]–[23]. Due to this evidence, we hypothesized that if C161, C229, and C379 are involved in the final structure of the capsid, it is due to early molecular interactions during capsid assembly and that disulfide bond formation does not take place between these residues in the mature capsid.

We found that after 10 and 20 days of tissue growth, C161S, C229S, and C379S virions produce lower viral titers compared to wild-type virions. Also, at both 10 and 20 days, C161S and C379S virions are less infectious than wild-type virions. They do not mature like wild-type virions, as they do not increase in infectivity between 10 and 20 days like wild-type. C229S virions are completely non-infectious. Further, C161S, C229S, and C379S mutant virions are less stable than wild-type virions. Our results suggest that C229 is important for DNA encapsidation and serves a critical, early stage role in capsid assembly, while C161 and C379 serve roles that may occur earlier or further downstream during capsid assembly that affect virion maturation. Our studies reveal differentiation-dependent roles for additional L1 cysteine residues other than inter-pentameric disulfide bonding and incorporate data from studies using recombinant papillomavirus capsids to explain the steps of capsid assembly and maturation in tissue.

## Results

### Establishment of Productive HPV16 C161S, C229S, and C379S Cell Lines

**Figure 1:**
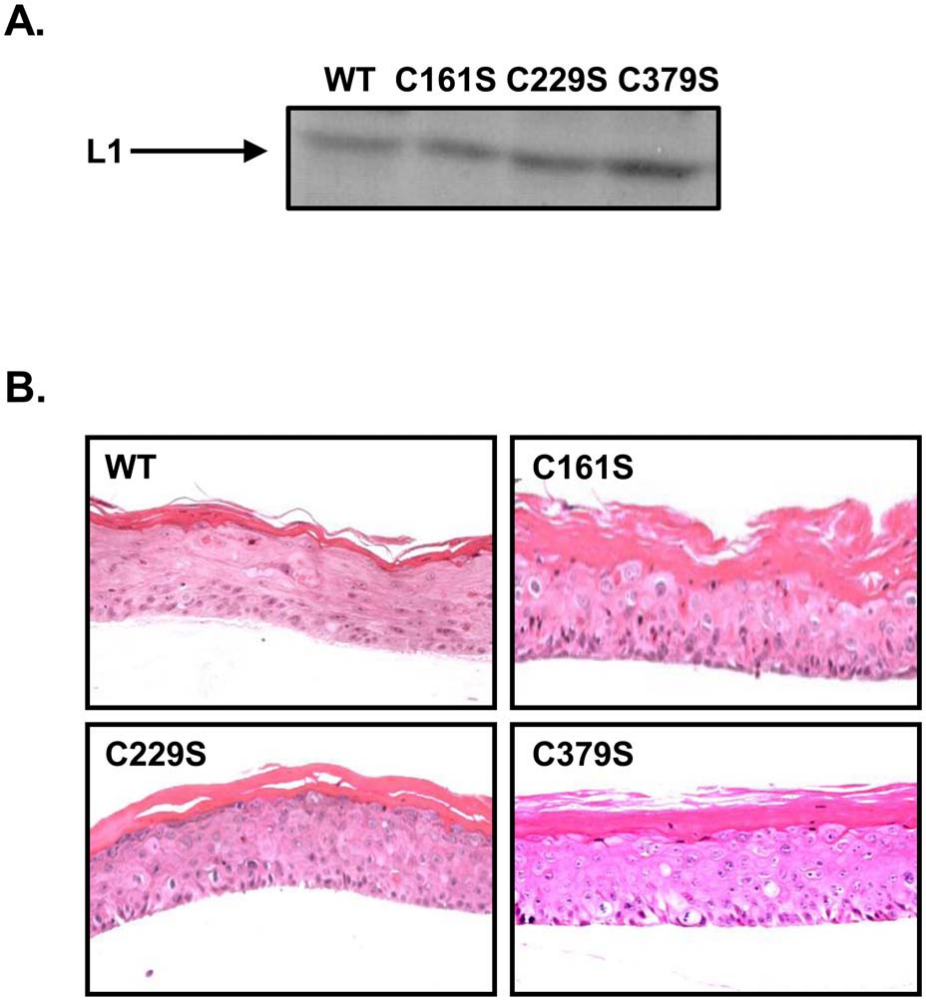
Mutations do not inhibit HPV16 L1 translation or organotypic raft growth. (**A**) Western blot of the major capsid protein, L1, from wild-type and mutant viral preps from 20-day rafts. Equal volumes of tissue extract were loaded for each sample. (**B**) Wild-type and mutant 20-day raft tissues were cut and stained with hematoxylin and eosin (H&E).

Productive cell lines that can synthesize HPV native virions in fully differentiating epithelial culture were described previously by our lab [15], [24]–[27]. Briefly, wild-type and mutant cell lines were created by electroporating primary human foreskin keratinocytes (HFKs) with linearized HPV16 wild-type or mutant full-length genomes and selecting for immortalized stable cell lines [15], [25]–[27]. Southern blots were performed to confirm the episomal nature of viral genomes in the productive cell lines and mutations in L1 were confirmed by DNA sequencing (data not shown). Once it was determined that the cell lines contained episomal viral DNA, they were grown in organotypic culture. Organotypic cultures were grown for 10 or 20 days in order to test both immature (10 days) and mature virions (20 days) [15]. All tissues containing mutant viral genomes produced L1 protein, stratified, and differentiated similar to tissues containing wild-type viral genomes (Fig. 1A–B). Mutations did not negatively affect viral genome replication during tissue growth as similar levels of genome amplification were detected for both 20-day wild-type and mutant virus-infected cells (Table 1).

**Table 1:** Amplification of wild-type and mutant viral genomes in 20-day differentiating tissue.

Virus Type	Fold Change in Amplification
Wild-type	9.60
C161S	10.20
C229S	9.21
C379S	9.83

### Viral Titers from 10- and 20-day HPV16 Wild-type and Mutant Tissues

It has been previously shown that C161S, C229S, and C379S mutations in HPV16 VLPs result in capsid instability, as mutant VLPs were more susceptible to tryptic proteolysis than wild-type [18]. A tentative conclusion was drawn that C161, C229, and C379 are involved in intra-pentameric disulfide bonding. However, cryo-EM structural data of BPV1 does not support disulfide bonding at these cysteines in a mature virion. Other viruses, though, like SV40, utilize cysteines for transient disulfide bonding early in capsid assembly.

To test whether C161S, C229S, and C379S substitutions affected steps in capsid assembly and maturation, we quantified the viral titers from 10- and 20-day tissues assessing the ability of wild-type and mutant L1 proteins to assemble and protect viral genomes from an endonuclease. Viral preps were treated with benzonase in order to eliminate exogenous viral genomes present within the lysate. Benzonase treatment has been previously reported to eliminate both chromatin-associated and chromatin-non-associated DNA, in addition to a large population of endonuclease-susceptible viral genomes, while leaving a population of protected viral genomes for downstream analysis [15], [16], [28].

**Figure 2:**
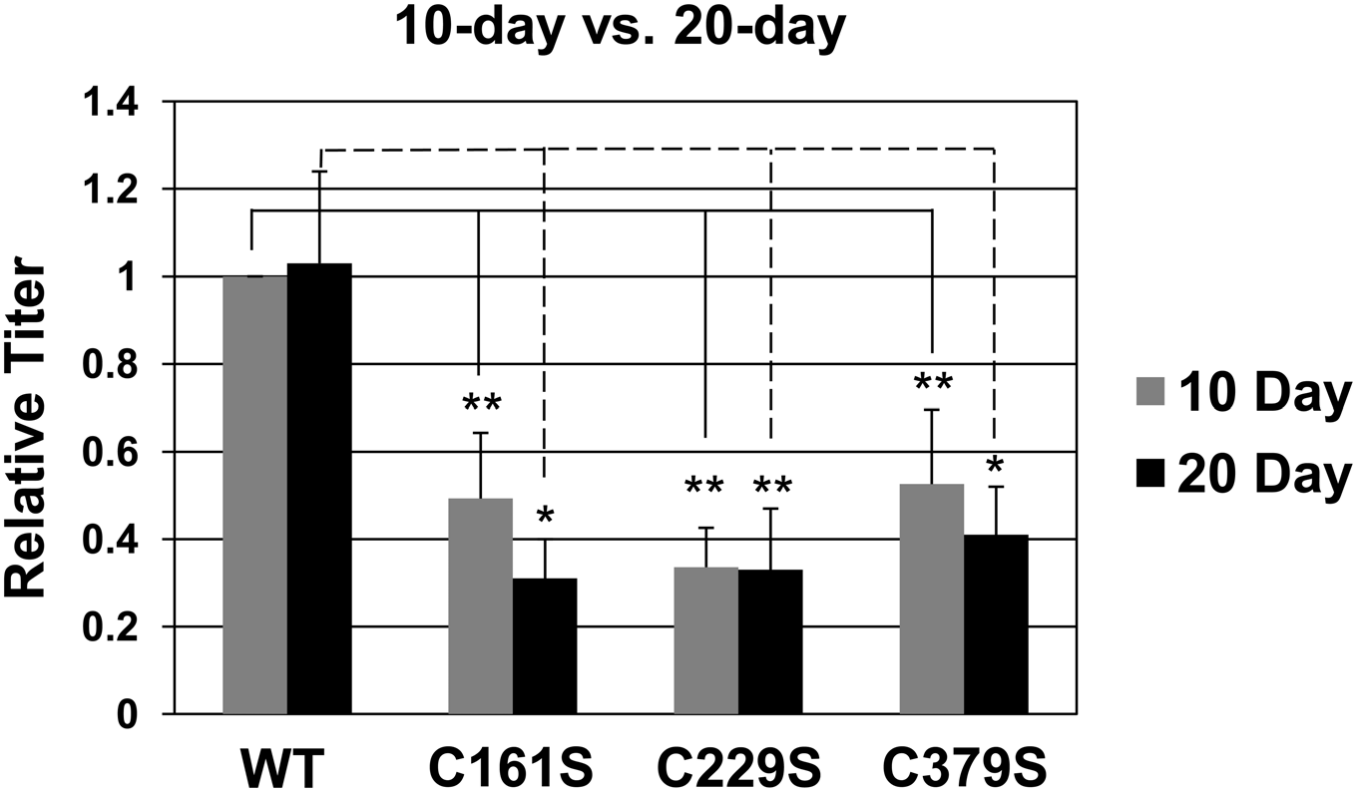
Mutation of HPV16 L1 C161, C229, and C379 reduce the efficiency of genome encapsidation. Relative viral titers from wild-type or mutant organotypic rafts at 10 and 20 days of growth were measured. Samples were first treated with benzonase to eliminate non-encapsidated genomes and were later quantified by amplifying a region of the viral E2 gene using qPCR.

10- and 20-day C161S, C229S, and C379S viral titers were 50% or less than 10- and 20-day wild-type titers (Fig. 2). This decrease in titers was observed even though within 20-day tissue viral genome replication occurred at an equivalent rate compared to wild-type virus-infected cells (Table 1). Titers did not change significantly between 10- and 20-day wild-type, C161S, C229S, and C379S viral preparations (Fig. 2). Endonuclease-resistant genomes in mutant viral preps compared to wild-type preps did not increase, which could suggest that the mutations at these cysteines cause early assembly problems, during the first 10 days, for the virus.

### Infectivity of 10- and 20-day HPV16 Wild-type and Mutant Virions

**Figure 3:**
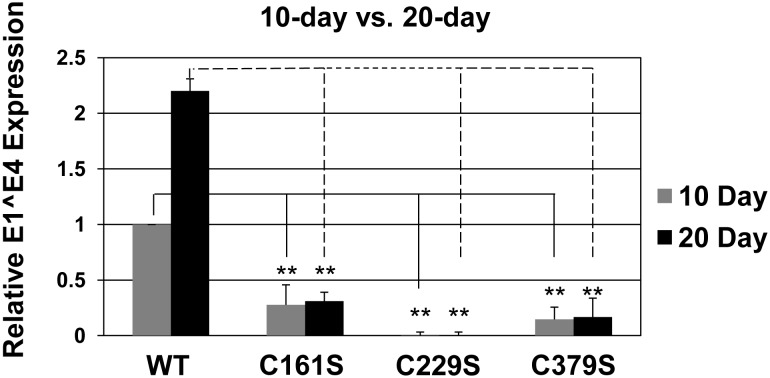
Mutation of HPV16 L1 C161, C229, and C379 negatively impact virus infectivity. Relative levels of the E1∧E4 spliced transcript were measured by RT-qPCR in HaCaT cells 48 hours post-infection at an equal MOI (MOI = 5) for virus harvested from both 10- and 20-day tissues.

Significant reductions in titers were detected in 10 and 20-day mutant viral preparations compared to wild-type (Fig. 2). However, detectable endonuclease-resistant genomes were found in all samples. The presence of these genomes suggested that, while inefficient, all of the mutant L1 capsids tested can assemble around and protect viral genomes from endonucleases. This supports data indicating these same cysteine to serine substitution mutants can produce viable VLPs as determined by electron microscopy [18]. To determine if mutant virions were infectious, an RT-qPCR-based infectivity assay that has been described previously was performed [15], [28]–[30]. Infections were performed at a MOI of 5. At both 10 and 20 days C161S and C379S mutant virus were less than 25% as infectious compared to wild-type virus (Fig. 3). The C229S mutant was non-infectious. We have previously shown that native HPV16 virions exploit a natural redox gradient that spans the human epithelium [15], [28]. This separation of reducing and oxidizing areas of the tissue facilitate virion assembly and maturation events in the context of the complete papillomavirus life cycle [15], [28]. One aspect of matured HPV16 wild-type virions is an increase in infectivity between virus collected from 10-day tissue and 20-day tissue, with 20-day tissue derived virus being more infectious [15]. Side-by-side comparison of 10- and 20-day viral preps shows that infectivity increases in 20-day wild-type virions compared to 10-day wild-type virions (Fig. 3). We had previously described some cysteine mutants designed to prevent inter-pentameric disulfide bonding (C175S and C185S) that increased in infectivity from 10 to 20 days of tissue growth. However, 20-day C161S and C379S mutant virus were no more infectious than 10-day mutant virus (Fig. 3). This indicates that mutations to either C161 or C379 prevent virions from achieving a mature HPV16 wild-type virion. As C229S is already non-infectious at 10 days, it suggests that C229 provides an essential step in assembly prior to the virion reaching a pre-mature state that is ultimately necessary for viral infectivity.

### Relative Stabilities of HPV16 Wild-type and Mutant Virions

**Figure 4:**
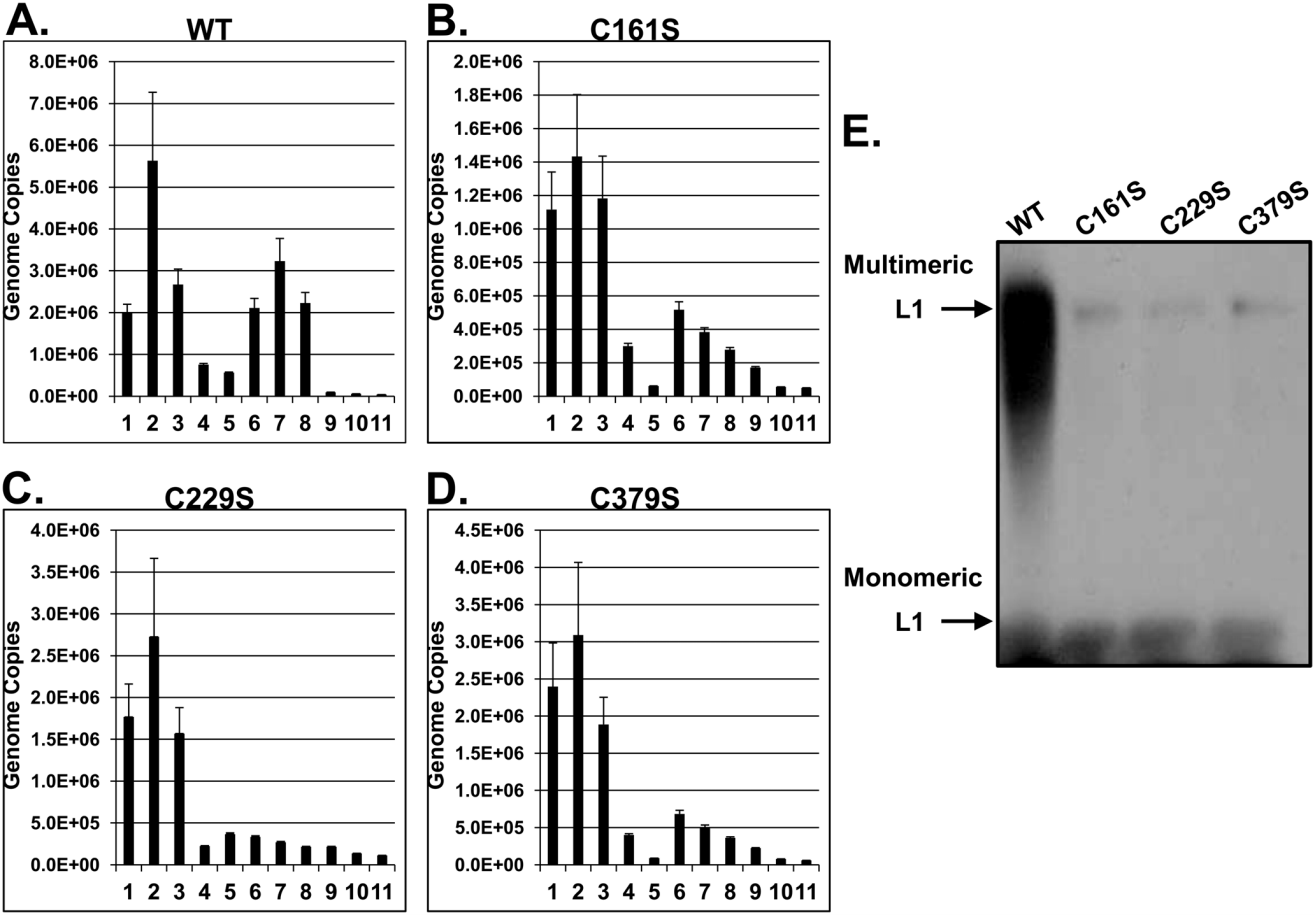
Mutation of HPV16 L1 C161, C229, and C379 causes capsid instability. (**A–D**) Total genome copies were measured after fractionating 20-day viral preps, previously treated with benzonase, in an ultracentrifuge. Fractions were not re-treated with benzonase. Equal volumes of each fraction were used to measure total genome copies per fraction. Genome copies were quantified by amplifying a region of the viral E2 gene using qPCR. (**E**) An L1 western blot was performed under non-reducing conditions to measure L1 multimer vs. monomer formation. Equal volumes of fraction #7 were analyzed for wild-type and each mutant.

We have previously shown that HPV mutants that have altered infectivity may also exhibit altered capsid stability [15], [18], [29]. For example, substituting a serine at C428, preventing the final inter-pentameric disulfide bond, causes virions to be 36% less infectious and 7.3 times less stable than wild-type virions [29]. We have already shown that the cysteines involved with inter-pentameric disulfide bonding within the capsid, C175, C185, and C428, to be fragile if replaced with serine [29]. Using C161S, C229S, and C379S mutant virus, we investigated their capsid assembly and stability by subjecting virions to Optiprep gradient ultracentrifugation, which has previously been reported to make viral genomes susceptible to endonuclease digestion if a capsid is unstable [15], [31]. After ultracentrifugation, 11 fractions were taken from the gradient (1 = top and 11 = bottom). Total genome copies were measured by qPCR within each fraction. As shown previously, fractions 1–4 contain genomes that are dissociated from their capsid as free genomes or are associated with disrupted capsids, as re-treatment of these fractions by an endonuclease digests these genomes [15]. Fractions 6–9 contain genomes that are still protected by their capsids and are therefore resistant to endonuclease treatment post-centrifugation [15]. HPV16 wild-type has a genome copy fractionation profile with 40% of its genomes detected in the most stable fractions, 6–9 (Fig. 4A). The C161S, C229S, and C379S mutants have different genome fractionation profiles, with less genomes detected in the stable fractions 6–9, at 24.3%, 10.3%, and 18.2% respectively (Fig. 4B–D). To quantify the differences, relative stabilities were calculated, defined as the ratio of total unprotected genomes to total protected genomes [29]. The lower the number, the more stable the population of virions. We have previously reported that the relative stability of HPV16 wild-type is approximately 1 [15]. Relative stabilities of the mutants were lower (Table 2). C161S, C229S, and C379S had relative stabilities of 3.0, 7.4, and 4.4, respectively. To determine the impact that these mutations have on multimeric formation of L1, purified virions from fraction #7 were also subjected to western blot under non-reducing conditions (Fig. 4E). We have previously shown that less multimeric L1 is detected in the stable fractions of purified virions containing mutations that impact particle stability compared to wild-type [29]. Again, we found that all three mutants have less multimeric L1 compared to wild-type. These data suggest that C161, C229, and C379 have a role during capsid assembly, which ultimately contributes to an increase in capsid stability.

**Table 2:** Relative stability values of 20-day wild-type and mutant virus.

Virus Type	Relative Stabilty
Wild-type	1.3
C161S	3.0
C229S	7.4
C379S	4.4

**Figure 5:**
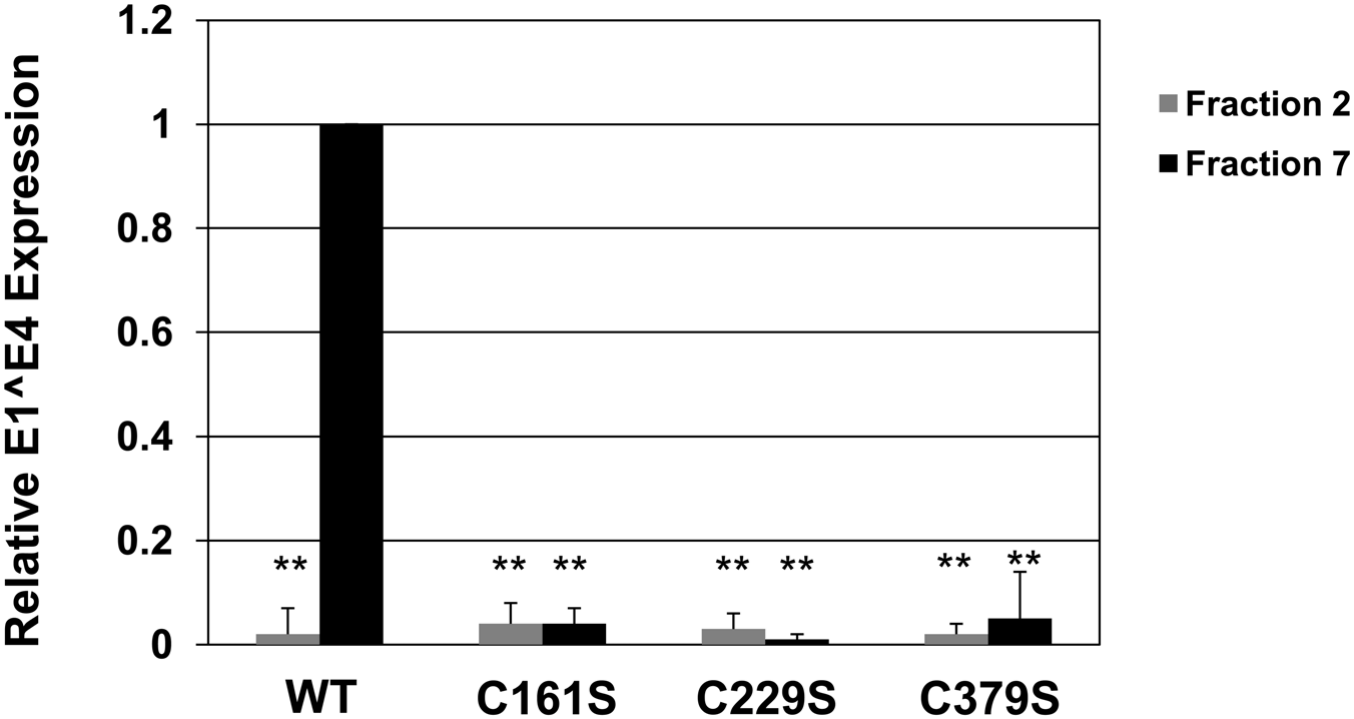
Mutation of HPV16 L1 C161, C229, and C379 reduce infectivity of virus from stable fractions. Relative levels (wild-type fraction #7 = 1) of the E1∧E4 spliced transcript were measured by RT-qPCR in HaCaT cells 48 hours post-infection at an equal MOI (MOI = 5) for fraction #2 and fraction #7 harvested from 20-day tissues. Fractions were not re-treated with benzonase.

To further examine the characteristics of the fractionated particles, total infectivity was analyzed for each fraction. Mature wild-type HPV16 virions have been shown to have most of their infectivity detected in the stable fractions, 6–9 [15] (S1). The 3 mutants had much lower infectivity than wild-type, with the C379S mutant being the most infectious (S1). As described previously, the infectivity detected in fractions 1–4 is generated from unprotected or free genomes [15]. Samples from wild-type and mutant fraction 2 and fraction 7 virions were used to compare infectivity at an equal MOI of 5 (Fig. 5). Mutant virions taken from fraction 7 were greatly reduced in their infectivity, with no mutant retaining more than 5% of wild-type infectivity. Samples of both wild-type and mutant virions from fraction 2 were not infectious relative to wild-type virions from fraction 7, with all samples yielding less than 4% infectivity. In summary, all mutants were found, at an equal MOI, to have much less infectivity within the stable fractions compared to wild-type virus. This suggests that the mutated viral capsids that migrate to the stable fractions are very inefficient at infecting their target cells.

## Discussion

HPV capsids depend upon strong disulfide bonds which form as the particles travel through a redox gradient that exists in human epithelium. Previous studies with HPV16 in monolayer culture using recombinant particles, as well as in differentiating tissue systems using native virus, have shown dependence on inter-pentameric disulfide bonding at C175, C185, and C428 [18], [29]. This study explored if three other cysteines, C161, C229, and C379, in HPV16 that are conserved among many HPV types contribute to virion assembly in differentiating tissue.

Previous experiments with VLPs have shown that capsids of these cysteine to serine mutants still appear structurally similar to wild-type capsids when viewed under EM [18]. In differentiating tissue, we found that the mutant genomes, C161S, C229S, and C379S, all produced lower viral titers at 10 and 20 days of tissue growth. This was unexpected, as stated previously, these same mutations form conformationally accurate VLPs when viewed under EM. It is possible that this highlights a role for these cysteines by the capsid for assembly within its natural environment, or that genome encapsidation utilizes these cysteines differently than if a VLP is formed around no genome.

It was also found that C161S and C379S virions are more than 50% less infectious than wild-type virus at both 10 and 20 days. C229S virions are non-infectious at either time point. These results support previous studies that suggest a role for C229 in intracellular trafficking during viral entry [32]. We have previously published that HPV16 wild-type virions undergo maturation within host tissue, utilizing a redox gradient as they pass up through the epithelium to a more oxidized environment [15]. Recombinant particles have also been shown to undergo structural changes when incubated in an oxidizing environment [33]. Redox also controls the rate of particle assembly [34]. Like many viruses, such as human immunodeficiency virus (HIV) and adenovirus, HPV16’s maturation has been linked to an increase in its infectivity, which occurs between 10- and 20-days in organotypic raft culture [15], [35], [36]. When comparing the infectivity of both C161S and C379 from 10- and 20-day tissue, no increase in infectivity was detected, suggesting that these mutations may have prohibited capsid maturation.

The relative stabilities of C161S, C229S, and C379S virions show they are more fragile than wild-type. C161S is the least affected mutant, but is still 3 times less stable than wild-type. This could have a severe impact on its ability to survive environmental stresses before it has a chance to make viral progeny. Even though these mutants have capsids that are less stable than wild-type, they have greater relative stabilities than those mutants impacting inter-pentameric disulfide bonding [29]. It should be noted that our stability assay cannot distinguish between L1 aggregates and virions, but previous neutralization assays using HPV16 wild-type from fraction 7 would suggest that a large majority are virions with correct conformation [15]. Also, aggregates appearing in fraction 7 would need to protect the genome from nuclease digestion and have the same density as native virions. In agreement with their relative stabilities, all three mutants had less detectable levels of multimeric L1, which we have previously shown to occur with mutations that affect disulfide bonding and capsid stability. It is possible that the amino acid change from cysteine to serine prevents assembly or maturation of the capsid from moving forward through improper protein conformation. Cryo-EM shows C161, C229, and C379 not being involved in the final disulfide bonding responsible for the integrity of the mature virion. Instead of being involved in stable inter-pentameric disulfide bonding, we suggest that these cysteines create transient disulfide bonds during early virion morphogenesis. SV40 has cysteines in its major capsid protein, VP1, that are responsible for inter-pentameric disulfide bonds as well as those that affect final capsid conformation either at early or late stages in assembly [21]–[23], [37], [38]. Identification of cysteines responsible for transient disulfide bonding is crucial to understanding the mechanism of papillomavirus assembly. This evidence highlights a potential shared evolutionary strategy between papillomaviruses and polyomaviruses to create a capsid stable enough to withstand environmental stress.

In conclusion, these data are consistent with a dynamic assembly process that is taking place within differentiated tissue by HPV16. It is possible that C229 is utilized sometime prior to the virion reaching a pre-mature state early in the assembly process (before 10 days), as it has the most negative impact on genome encapsidation, infectivity, and capsid stability if mutated to serine. Then, as the virion matures (after 10 days), the virion uses C161 and C379, because mutation of these cysteines appear to cause a block at the step of maturation. It is also possible that substituting C161 and C379 for serine hinders an early assembly step, however less critical than the step requiring C229, thus causing a smaller negative effect on the later steps of capsid assembly. As shown by their relative stability numbers, mutation of C161, C229, and C379 do not impact the structural integrity of the virion as much as mutants of inter-pentameric disulfide bonding. However, these cysteines are conserved among many HPV types and help orchestrate the assembly and maturation process of the particle to its final conformation within differentiated tissue.

## Materials and Methods

### Ethics Statement

The use of human foreskin keratinocyte tissues to develop cell lines for these studies was approved by the Institutional Review Board at the Pennsylvania State University College of Medicine and by the Institutional Review Board at Pinnacle Health Hospitals. Discarded tissues were exempt from needing informed patient consent. Informed consent was waived by both Institutional Review Boards. No identifiers were attached to any tissue samples.

### Mutagenesis

pBSHPV16 (114/B) DNA, a generous gift from M. Dürst, was utilized as the template for site-directed mutagenesis using Strategene’s Quikchange II XL Site-Directed Mutagenesis Kit as described previously [29]. Multiple mutant viral genomic clones containing correctly mutagenized sequences were isolated per each mutation and utilized in subsequent experimentation. To create a full-length HPV16 (114/B) genome with a C379S substitution, the two complimentary oligonucleotides forward 5′ CAGTTTATTTTTCAACTGTCTAAAATAACCTTAACTGCAG 3′ and reverse 5′ CTGCAGTTAAGGTTATTTTAGACAGTTGAAAAATAAACTG 3′, changed GC to CT at nucleotides 6772–6773. To create a full-length HPV16 (114/B) genome with a C161S substitution, the two complimentary oligonucleotides forward 5′ GTGTTTAATTGGTTCTAAACCACCTATAGGGGAACAC 3′ and reverse 5′ GTGTTCCCCTATAGGTGGTTTAGAACCAATTAAACAC 3′, changed GC to CT at nucleotides 6119–6120. To create a full-length HPV16 (114/B) genome with a C229S substitution, the two complimentary oligonucleotides forward 5′ GATATTTGTACATCTATTTCTAAATATCCAG 3′ and reverse 5′ CTGGATATTTAGAAATAGATGTACAAATATC 3′, changed GC to CT at nucleotides 6323–6324. In all instances, the most prevalent serine codon in the HPV16 (114/B) L1 ORF, TCT, was utilized.

### Keratinocyte Cultures and Electroporation

HFKs were isolated and grown from newborn circumcision as described previously [26]. For electroporations, 30 µg of the mutated viral DNA was digested with BamHI, linearizing the viral DNA and separating it from the vector sequence. HFKs were electroporated with the prepared DNA as described previously [24], [26]. Multiple cell lines were obtained for each mutated genome. The mutation region of the viral genome was verified by sequencing for each cell line used.

### Southern Blot Hybridization

Total cellular DNA was isolated as previously described [26]. Briefly, 5 µg of total cellular DNA was digested with BamHI, linearizing the HPV16 genome. Samples were separated by 0.8% agarose gel electrophoresis and transferred onto a GeneScreen Plus membrane (New England Nuclear Research Products) as previously described [39]. Hybridization of the membrane used an HPV16-specific, whole-genome probe as previously described [26], [39].

### Organotypic Raft Culture-derived Virion Production

Immortalized HFK lines, with stably maintained episomal HPV16 DNA, were grown in monolayer culture using E-medium in the presence of mitomycin C-treated J2 3T3 feeder cells [26]. Raft tissues were grown for 20 days as previously described [15], [26].

### Histology

Tissues were fixed in 10% neutral buffered formalin and embedded in paraffin. Sections were cut and stained with hematoxylin and eosin (H&E) as previously described [24]. At least two different batches of organotypic tissues were used for each virus type.

### HPV Isolation

For Optiprep (Sigma-Aldrich) fractionation, quantitative RT-PCR (RT-qPCR) and qPCR-based DNA titering assays were used. To obtain virus preps, three rafts were prepared by dounce homogenization in 500 µl of phosphate buffer (0.05 M sodium phosphate [pH 8.0], 2 mM MgCl_2_). Homogenizers were rinsed with 250 µl of phosphate buffer. Then, 1.5 µl (375 U) of benzonase was added to 750 µl of virus prep, followed by incubation at 37°C for 1 hour. Samples were brought to 1 M NaCl by added 195 µl of 5 M NaCl. Then, samples were mixed and centrifuged at 4°C for 10 minutes at 10,500 rpm in a microcentrifuge. The supernatants were stored at −20°C.

### Optiprep Purification of Virions

Optiprep purification was performed as described previously [15], [31]. Briefly, 27%, 33%, and 39% Optiprep gradients were produced by underlayering. Gradients were allowed to diffuse for 1 hour at room temperature. Then, approximately 350 µl of benzonase-treated virus prep was layered on top of the gradient. Tubes were centrifuged in a SW55 rotor (Beckman) at 234,000×*g* for 3.5 hours at 16°C. Post centrifugation, 11, 500 µl fractions were carefully collected starting from the top of each tube (top = 1 and bottom = 11).

### qPCR-based DNA Titering Assay

To detect endonuclease-resistant genomes in viral preps or Optiprep fractions, only benzonase-treated viral preps were utilized so that all non-encapsidated genomes were digested [15], [28], [29]. To release all encapsidated viral genomes, 10 µl of viral prep or Optiprep fraction was added to 2 µl of proteinase K, 10 µl of 10% sodium dodecyl sulfate, and brought up to 200 µl with Hirt buffer. Tubes were rotated at 37°C for 2 hours. Immediately, an equal amount of phenol-chloroform-isoamyl alcohol (25∶24∶1) was added and the mixture was extracted for the aqueous phase. An equal amount of chloroform was added and again extracted for the aqueous phase. DNA was ethanol precipitated overnight at −20°C. After centrifugation, the DNA pellet was washed with 70% ethanol and resuspended in 20 µl of Tris-EDTA overnight. To detect viral genomes, a SYBR green PCR kit (Bio-Rad) was utilized. Amplification of the viral target was performed in 0.2 ml, 96-well PCR plates (Bio-Rad) with a total reaction volume of 25 µl. Then, 1 µl of each endonuclease-resistant viral genome prep was analyzed in duplicate for each independent experiment. Amplification of HPV16 genomes was performed using primers complimentary to the HPV16 E2 gene: forward 5′ CCATATAGACTATTGGAAACACATGCGCC 3′ and reverse 5′ CGTTAGTTGCAGTTCAATTGCTTGTAATGC 3′. Oligonucleotides were synthesized by Integrated DNA Technologies (Coralville, IA). A standard curve was generated by amplifying 1 µl aliquots of 10^8^, 10^7^, 10^6^, 10^5^, 10^4^, and 10^3^ serially diluted pBSHPV16 copy number controls. Acceptable R^2^ values for standard curves were at or above 0.99. A Bio-Rad CFX-96 Real-Time qPCR machine and software were utilized for PCR amplifications and subsequent data analysis. The PCR thermocycling profile was as follows: a 15-minute hot-start at 95°C, followed by 40 cycles at 15 seconds at 94°C, 30 seconds at 52°C, and 30 seconds at 72°C. The data was collected during the extension phase. Results are representative of means and standard deviations for at least three independent infections from at least 2 different batches of virus preps for each virus. Student’s t-test was performed with statistical significance calculated (p<0.05).

### RT-qPCR Infectivity Assays

HaCaT cells were grown to confluence in Dulbecco modified Eagle medium supplemented with 10% fetal bovine serum, 2 mM glutamine, 1 mM pyruvate, 100 U of penicillin/ml, and 100 µg of streptomycin/ml and seeded 50,000 cells/well in 24-well plates [15], [28], [29]. HPV16 viral preps were diluted with cell culture medium to a total volume of 0.5 ml. Medium was aspirated from HaCaT cells, and 0.5 ml of diluted viral prep was added per well. An MOI of 5 for non-fractionated virus was utilized for infections. One well on each plate received 0.5 ml of medium without virus as a negative control. The cells were incubated with the virus for 48 hours at 37°C. mRNA was harvested with the RNeasy kit (Qiagen). Amplification of both the viral target and endogenous cellular control target was performed by using a duplex format in 0.2 ml, 96-well PCR plates (Bio-Rad). All reactions containing RNAs from virus-infected cells were performed in duplicate. RT-qPCR was performed in the same closed tube with ∼250 ng of total RNA per reaction using the Quantitect probe RT-PCR kit (Qiagen). The HPV16 E1∧E4 primers used were: forward 5′ GCTGATCCTGCAAGCAACGAAGTATC 3′ and reverse 5′ TTCTTCGGTGCCCAAGGC 3′ at final concentrations of 4 µM. A fluorogenic, dual-labeled, HPV16 E1∧E4 probe (5′-6-FAM-CCCGCCGCGACCCATACCAAAGCC-BHQ-1-3′) was utilized at a final concentration of 0.2 µM to detect E1∧E4 cDNA. TATA-binding protein (TBP) was amplified as an internal control using the primers: 5′-CACGGCACTGATTTTCAGTTCT-3′ and 5′-TTCTTGCTGCCAGTCTGGACT-3′ at final concentrations of 0.125 µM. A fluorogenic, dual labeled, TBP probe (5′-5-HEX-TGTGCACAGGAGCCAAGAGTGAAGA-BHQ-1-3′) was utilized at a final concentration of 0.3 µM to detect TBP cDNA. All primers were synthesized by Integrated DNA Technologies (Coralville, IA). All RT-qPCRs were performed using the CFX-96 (Bio-Rad). Cycling conditions were 50°C for 30 minutes (RT) and 95°C for 15 seconds, followed by 42 cycles of 94°C for 15 seconds and 54.5°C for 1 minute. The relative quantities of viral target cDNA were determined by using REST software. Results are representative of means and standard deviations for at least three independent infections from at least 2 different batches of virus preps for each virus type. Student’s t-test was performed with statistical significance calculated (p<0.05).

### Immunoblot Analysis

Equal volume aliquots from organotypic tissue extracts were boiled for 10 minutes in 6% 2 mercaptoethanol (2-ME) loading buffer and loaded onto a 7.5% polyacrylamide gel. Nitrocellulose membranes were blocked using StartingBlock blocking buffer in PBST (Thermo Scientific). To detect HPV16 L1, the anti-HPV16 L1 monoclonal antibody Camvir-1 (BD Pharmigen) was utilized at a 1∶2,000 dilution. An HRP-linked sheep anti-mouse secondary antibody was utilized at a 1∶8,000 dilution. Membranes were washed with PBST after the addition of each antibody. All antibodies were diluted in StartingBlock (Thermo Scientific). HRP was detected using an ECL kit (Perkin Elmer). The conditions for non-reducing L1 immunoblots have been previously described [29]. Results are representative of at least 2 different batches of virus preps for each virus type.

## Supporting Information

Figure S1 **Mutation of HPV16 L1 C161, C229, and C379 changes the infectivity profile of fractionated virus.** Relative infectivity (wild-type fraction #7 = 1) was measured after fractionating 20-day viral preps, previously treated with benzonase, in an ultracentrifuge. Fractions were not re-treated with benzonase. Equal volumes of each fraction were used to measure relative infectivity per fraction by detecting the E1∧E4 spliced transcript using RT-qPCR in HaCat cells 48 hours post-infection.
(TIF)

## References

[1] Buck CB, Cheng N, Thompson CD, Lowy DR, Steven AC, et al (2008) Arrangement of L2 within the papillomavirus capsid. J Virol 82: 5190–5197. 18367526 10.1128/JVI.02726-07 PMC2395198

[2] Kirnbauer R, Booy F, Cheng N, Lowy DR, Schiller JT (1992) Papillomavirus L1 major capsid protein self-assembles into virus-like particles that are highly immunogenic. Proc Natl Acad Sci U S A 89: 12180–12184. 1334560 10.1073/pnas.89.24.12180 PMC50722

[3] Kirnbauer R, Taub J, Greenstone H, Roden R, Durst M, et al (1993) Efficient self-assembly of human papillomavirus type 16 L1 and L1–L2 into virus-like particles. J Virol 67: 6929–6936. 8230414 10.1128/jvi.67.12.6929-6936.1993 PMC238150

[4] Trus BL, Roden RB, Greenstone HL, Vrhel M, Schiller JT, et al (1997) Novel structural features of bovine papillomavirus capsid revealed by a three-dimensional reconstruction to 9 A resolution. Nat Struct Biol 4: 413–420. 9145113 10.1038/nsb0597-413

[5] Baker TS, Drak J, Bina M (1989) The capsid of small papova viruses contains 72 pentameric capsomeres: direct evidence from cryo-electron-microscopy of simian virus 40. Biophys J 55: 243–253. 2540847 10.1016/S0006-3495(89)82799-7 PMC1330465

[6] Baker TS, Newcomb WW, Olson NH, Cowsert LM, Olson C, et al (1991) Structures of bovine and human papillomaviruses. Analysis by cryoelectron microscopy and three-dimensional image reconstruction. Biophys J 60: 1445–1456. 1663794 10.1016/S0006-3495(91)82181-6 PMC1260204

[7] Belnap DM, Olson NH, Cladel NM, Newcomb WW, Brown JC, et al (1996) Conserved features in papillomavirus and polyomavirus capsids. J Mol Biol 259: 249–263. 8656427 10.1006/jmbi.1996.0317 PMC4142696

[8] Finnen RL, Erickson KD, Chen XS, Garcea RL (2003) Interactions between papillomavirus L1 and L2 capsid proteins. J Virol 77: 4818–4826. 12663788 10.1128/JVI.77.8.4818-4826.2003 PMC152166

[9] Culp TD, Cladel NM, Balogh KK, Budgeon LR, Mejia AF, et al (2006) Papillomavirus particles assembled in 293TT cells are infectious in vivo. J Virol 80: 11381–11384. 16943284 10.1128/JVI.01328-06 PMC1642142

[10] Modis Y, Trus BL, Harrison SC (2002) Atomic model of the papillomavirus capsid. Embo j 21: 4754–4762. 12234916 10.1093/emboj/cdf494 PMC126290

[11] Ishii Y, Ozaki S, Tanaka K, Kanda T (2005) Human papillomavirus 16 minor capsid protein L2 helps capsomeres assemble independently of intercapsomeric disulfide bonding. Virus Genes 31: 321–328. 16175337 10.1007/s11262-005-3250-3

[12] Sapp M, Fligge C, Petzak I, Harris JR, Streeck RE (1998) Papillomavirus assembly requires trimerization of the major capsid protein by disulfides between two highly conserved cysteines. J Virol 72: 6186–6189. 9621087 10.1128/jvi.72.7.6186-6189.1998 PMC110432

[13] Li M, Beard P, Estes PA, Lyon MK, Garcea RL (1998) Intercapsomeric disulfide bonds in papillomavirus assembly and disassembly. J Virol 72: 2160–2167. 9499072 10.1128/jvi.72.3.2160-2167.1998 PMC109511

[14] Fligge C, Schafer F, Selinka HC, Sapp C, Sapp M (2001) DNA-induced structural changes in the papillomavirus capsid. J Virol 75: 7727–7731. 11462046 10.1128/JVI.75.16.7727-7731.2001 PMC115009

[15] Conway MJ, Alam S, Ryndock EJ, Cruz L, Christensen ND, et al (2009) Tissue-spanning redox gradient-dependent assembly of native human papillomavirus type 16 virions. J Virol 83: 10515–10526. 19656879 10.1128/JVI.00731-09 PMC2753102

[16] Conway MJ, Meyers C (2009) Replication and assembly of human papillomaviruses. J Dent Res 88: 307–317. 19407149 10.1177/0022034509333446 PMC3317948

[17] Conway MJ, Cruz L, Alam S, Christensen ND, Meyers C (2011) Cross-neutralization potential of native human papillomavirus N-terminal L2 epitopes. PLoS One 6: e16405. 21346798 10.1371/journal.pone.0016405 PMC3035607

[18] Ishii Y, Tanaka K, Kanda T (2003) Mutational analysis of human papillomavirus type 16 major capsid protein L1: the cysteines affecting the intermolecular bonding and structure of L1-capsids. Virology 308: 128–136. 12706096 10.1016/s0042-6822(02)00099-5

[19] Chen XS, Garcea RL, Goldberg I, Casini G, Harrison SC (2000) Structure of small virus-like particles assembled from the L1 protein of human papillomavirus 16. Mol Cell 5: 557–567. 10882140 10.1016/s1097-2765(00)80449-9

[20] Wolf M, Garcea RL, Grigorieff N, Harrison SC (2010) Subunit interactions in bovine papillomavirus. Proc Natl Acad Sci U S A 107: 6298–6303. 20308582 10.1073/pnas.0914604107 PMC2852008

[21] Li PP, Nakanishi A, Fontanes V, Kasamatsu H (2005) Pairs of Vp1 cysteine residues essential for simian virus 40 infection. J Virol 79: 3859–3864. 15731281 10.1128/JVI.79.6.3859-3864.2005 PMC1075729

[22] Gharakhanian E, Mana W, Norng M (2005) Cys254 and Cys49/Cys87of simian virus 40 Vp1 are essential in formation of infectious virions. Virus Res 107: 21–25. 15567029 10.1016/j.virusres.2004.06.006

[23] Li PP, Nakanishi A, Clark SW, Kasamatsu H (2002) Formation of transitory intrachain and interchain disulfide bonds accompanies the folding and oligomerization of simian virus 40 Vp1 in the cytoplasm. Proc Natl Acad Sci U S A 99: 1353–1358. 11805304 10.1073/pnas.032668699 PMC122194

[24] Meyers C, Frattini MG, Hudson JB, Laimins LA (1992) Biosynthesis of human papillomavirus from a continuous cell line upon epithelial differentiation. Science 257: 971–973. 1323879 10.1126/science.1323879

[25] McLaughlin-Drubin ME, Christensen ND, Meyers C (2004) Propagation, infection, and neutralization of authentic HPV16 virus. Virology 322: 213–219. 15110519 10.1016/j.virol.2004.02.011

[26] McLaughlin-Drubin ME, Wilson S, Mullikin B, Suzich J, Meyers C (2003) Human papillomavirus type 45 propagation, infection, and neutralization. Virology 312: 1–7. 12890615 10.1016/s0042-6822(03)00312-x

[27] Meyers C, Mayer TJ, Ozbun MA (1997) Synthesis of infectious human papillomavirus type 18 in differentiating epithelium transfected with viral DNA. J Virol 71: 7381–7386. 9311816 10.1128/jvi.71.10.7381-7386.1997 PMC192083

[28] Conway MJ, Alam S, Christensen ND, Meyers C (2009) Overlapping and independent structural roles for human papillomavirus type 16 L2 conserved cysteines. Virology 393: 295–303. 19733888 10.1016/j.virol.2009.08.010 PMC2763999

[29] Conway MJ, Cruz L, Alam S, Christensen ND, Meyers C (2011) Differentiation-dependent interpentameric disulfide bond stabilizes native human papillomavirus type 16. PLoS One 6: e22427. 21811610 10.1371/journal.pone.0022427 PMC3139651

[30] Cruz L, Meyers C (2013) Differential dependence on host cell glycosaminoglycans for infection of epithelial cells by high-risk HPV types. PLoS One 8: e68379. 23861898 10.1371/journal.pone.0068379 PMC3701689

[31] Buck CB, Pastrana DV, Lowy DR, Schiller JT (2004) Efficient intracellular assembly of papillomaviral vectors. J Virol 78: 751–757. 14694107 10.1128/JVI.78.2.751-757.2004 PMC368835

[32] Ishii Y, Kondo K, Matsumoto T, Tanaka K, Shinkai-Ouchi F, et al (2007) Thiol-reactive reagents inhibits intracellular trafficking of human papillomavirus type 16 pseudovirions by binding to cysteine residues of major capsid protein L1. Virol J 4: 110. 17961263 10.1186/1743-422X-4-110 PMC2147014

[33] Buck CB, Thompson CD, Pang YY, Lowy DR, Schiller JT (2005) Maturation of papillomavirus capsids. J Virol 79: 2839–2846. 15709003 10.1128/JVI.79.5.2839-2846.2005 PMC548454

[34] Mukherjee S, Thorsteinsson MV, Johnston LB, DePhillips PA, Zlotnick A (2008) A quantitative description of in vitro assembly of human papillomavirus 16 virus-like particles. J Mol Biol 381: 229–237. 18585738 10.1016/j.jmb.2008.05.079

[35] Joshi A, Nagashima K, Freed EO (2006) Mutation of dileucine-like motifs in the human immunodeficiency virus type 1 capsid disrupts virus assembly, gag-gag interactions, gag-membrane binding, and virion maturation. J Virol 80: 7939–7951. 16873251 10.1128/JVI.00355-06 PMC1563813

[36] Perez-Berna AJ, Ortega-Esteban A, Menendez-Conejero R, Winkler DC, Menendez M, et al (2012) The role of capsid maturation on adenovirus priming for sequential uncoating. J Biol Chem 287: 31582–31595. 22791715 10.1074/jbc.M112.389957 PMC3438990

[37] Jao CC, Weidman MK, Perez AR, Gharakhanian E (1999) Cys9, Cys104 and Cys207 of simian virus 40 Vp1 are essential for inter-pentamer disulfide-linkage and stabilization in cell-free lysates. J Gen Virol 80 (Pt 9): 2481–2489. 10.1099/0022-1317-80-9-2481 10501505

[38] Li PP, Nakanishi A, Tran MA, Salazar AM, Liddington RC, et al (2000) Role of simian virus 40 Vp1 cysteines in virion infectivity. J Virol 74: 11388–11393. 11070039 10.1128/jvi.74.23.11388-11393.2000 PMC113244

[39] Ozbun MA, Meyers C (1998) Human papillomavirus type 31b E1 and E2 transcript expression correlates with vegetative viral genome amplification. Virology 248: 218–230. 9721231 10.1006/viro.1998.9285 PMC3600430

