# Supplementary figures and images for: Roles for Human Papillomavirus Type 16 L1 Cysteine Residues 161, 229, and 379 in Genome Encapsidation and Capsid Stability
Source: PLoS One. 2014 Jun 11;9(6):e99488. doi: 10.1371/journal.pone.0099488 (PMC4053435; doi:10.1371/journal.pone.0099488)

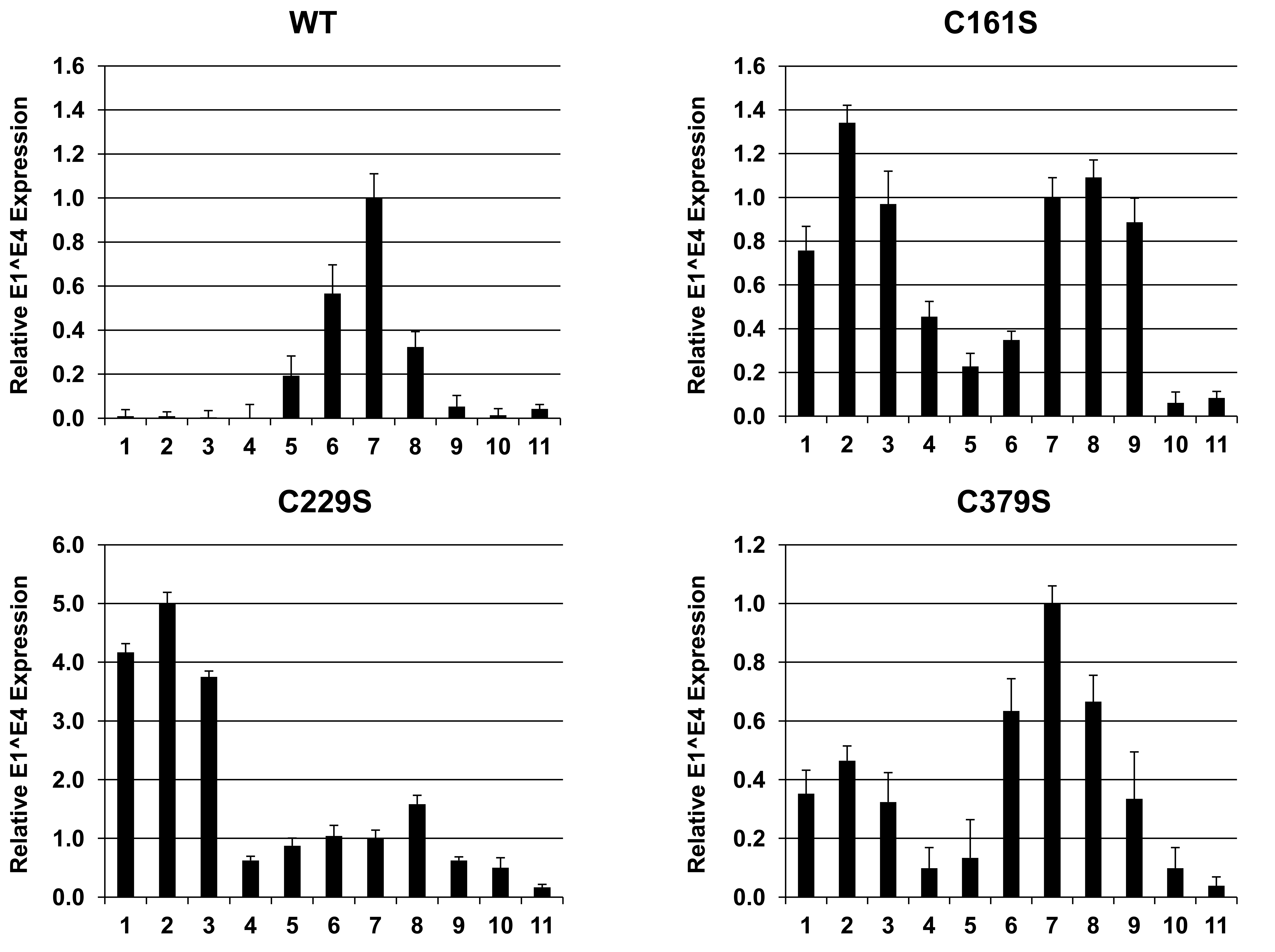

Supplement: Figure S1 — Mutation of HPV16 L1 C161, C229, and C379 changes the infectivity profile of fractionated virus. Relative infectivity (wild-type fraction #7 = 1) was measured after fractionating 20-day viral preps, previously treated with benzonase, in an ultracentrifuge. Fractions were not re-treated with benzonase. Equal volumes of each fraction were used to measure relative infectivity per fraction by detecting the E1∧E4 spliced transcript using RT-qPCR in HaCat cells 48 hours post-infection. (TIF) [file pone.0099488.s001.tif]
